# Transitive reasoning in the adult domestic hen in a six-term series task

**DOI:** 10.1007/s10071-024-01914-1

**Published:** 2024-11-19

**Authors:** R. Degrande, O. Amichaud, B. Piégu, F. Cornilleau, P. Jardat, V. H.B. Ferreira, V. Colson, L. Lansade, L. Calandreau

**Affiliations:** 1 CNRS, INRAE, Université de Tours, PRC (Physiologie de la Reproduction et des Comportements), Nouzilly, Indre-et-Loire F-37380 France; 2 Laboratoire de Physiologie et Génomique des Poissons, INRAE, Rennes, France; 3 IFIP - Institut du Porc, 9 Boulevard du Trieux, Pacé, 35740 France

**Keywords:** Transitive inference, Relational memory, Cognition, Chicken, *Gallus gallus Domesticus*

## Abstract

Transitive inference (TI) is a disjunctive syllogism that allows an individual to indirectly infer a relationship between two components, by knowing their respective relationship to a third component (if A > B and B > C, then A > C). The common procedure is the 5-term series task, in which individuals are tested on indirect, unlearned relations. Few bird species have been tested for TI to date, which limits our knowledge of the phylogenetic spread of such reasoning ability. Here we tested TI in adult laying hens using a more solid methodology, the 6-term series task, which has not been tested in poultry so far. Six hens were trained to learn direct relationships in a sequence of six arbitrary items (A > B > C > D > E > F) in a hybrid training procedure. Then, 12 testing sessions were run, comprising 3 non-rewarded inference trials each: BD, BE, and CE. All subjects showed TI within 12 inference trials and were capable of TI whatever the relative distance between the items in the series. We found that TI performance was not impacted by the reinforcement ratios of the items for most individuals, making it harder to support a purely associative-based resolution of the task. We suggest that TI is based on the same cognitive processes in poultry (*Galloanserae*) than in modern flying birds (*Neoaves*), and that the cognitive strategy to solve the task might be driven mainly by individual parameters within species. These results contribute to a better understanding of transitive inference processes in birds.

**Supplementary Information:**

The online version contains supplementary material available at 10.1007/s10071-024-01914-1.

## Introduction

To adapt efficiently to their living environment, animals generally use specific cognitive capacities, which may depend on species-specific physical or social constraints. In large groups, the ability to recognize conspecifics and to position themselves in the hierarchy enables individuals to avoid potential injuries due to predictable conflicts (Zuberbühler and Byrne 2006; Croney and Newberry 2007). In free-living conditions, hens form social groups organized according to a strict linear hierarchy, maintained by displays of dominance, also known as the “pecking order” (Craig 1978). They are known to use a wide range of socio-cognitive skills to navigate such a complex social structure. For example, hens are able to recognize their peers based on several individual features (Abeyesinghe et al. 2009), and to adapt their behavior towards an unknown individual, depending on whether this individual won or lost the interaction with a known, dominant individual (Hogue et al. 1996).

Among these socio-cognitive capacities, transitive inference (TI) is the ability to indirectly infer the relationship between two individuals, by knowing their respective relationship with a third individual. For example, if individual A is dominant over individual B, and individual B is dominant over individual C, then individual C can infer that individual A is dominant over themself. TI is thus particularly useful in hierarchical-group living. This complex ability requires a long-term memory about the relationships (relational memory) and the ability to use this knowledge to infer the indirect relationships (Cohen and Eichenbaum 1993; Guez and Audley 2013).

The common task to test TI in animals is the five-term series task (Piaget 1928; Bryant and Trabasso 1971; McGonigle and Chalmers 1977; Aust et al. 2008; Mikolasch et al. 2013). In this task, the individuals have to learn dyadic relationships between successive items in a five-term ordered sequence A > B > C > D > E (where the letters stand for the different items). That is, for example, when faced with the items A and B, individuals are reinforced when choosing A; when faced with B and C, they are reinforced when choosing B, and so on. Then, individuals are tested on the unanticipated, nonadjacent pair BD. Choosing B over D is interpreted as a demonstration of TI, as B and D are indirectly linked through their relationship with the third item C (learned relationships: B > C and C > D). However, the use of the five-term series task has been questioned in the literature (Allen 2006). Since in a five-term series, B is directly linked to A, the most positive item in terms of probability of reinforcement, and D is directly linked to E, the most negative item, this configuration impedes discussions about the cognitive process at stake both during relational learning and during TI.

In the present study, we adapted a six-term series task to adult hens (Gallus gallus domesticus), with a twofold objective. The first one was to use a larger sequence of items, involving three non-adjacent and non-endpoint pairs (BD, BE and CE) allowing for the drawing of more solid conclusions about TI processes in this species. Notably, we aimed at questioning whether hens base their transitive responding solely on associative values, or whether they can show inference, through making indirect relations from the direct relations they learned. This approach has already been successfully used in other avian species, such as pigeons and corvids (von Fersen et al. 1991; Bond et al. 2003; Daniels et al. 2014). The second one was to broaden our knowledge of the cognitive abilities of domesticated birds compared to non-domesticated species, since the former are generally expected to be less capable due to artificial selection. (Ferreira et al. 2023).

Hens were trained following a procedure adapted from the hybrid training procedure in pigeons, as proposed by Daniels et al. (2014). We ran a six-term series of arbitrary items of different shapes and colors. Pairs were trained successively (AB, BC, CD, DE and EF), and mixed sessions, containing each previously trained pair, were included at each step. Such a hybrid training procedure, by gradually increasing complexity and by keeping showing every pair throughout the whole training stage, allows to maximize and equalize the performance for every pair trained and to avoid different delays between the last time each pair has been trained and test sessions (Vasconcelos 2008; Daniels et al. 2014). This six-term series task allowed us to test three different inference trials: BD, BE and CE. As already mentioned, instead of relying solely on a single pairwise comparison, these three trials allow for complementary analyses and more robust results (Vasconcelos 2008; Bond et al. 2010).

We analyzed the learning performance, the TI performance and the choice latency for each adjacent pair (premise pairs) and for each nonadjacent pair (control and inference trials, respectively), at the group level and at the individual level, to measure eventual inter-individual variations. We put an emphasis on the learning accuracy between the premise pairs as it might give some indications about the cognitive processes in the TI task (Bond et al. 2010). Finally, we tested for alternative cues that could have driven the choice behaviour of individuals through the configuration of the sessions.

## Method

### Ethics approval

This experimental procedure was approved by the Val de Loire Ethics Committee (approval n° CE19–2021-0211-1, CEEA VdL, France). Animal care and experimental treatments complied with the French and European guidelines for housing and care of animals used for scientific purposes (European Union Directives 2010/63/AU).

### Subjects and housing

Six adult laying hens (Isa Brown strain), aged 2 to 3 years old, were included in the procedure. The hens were maintained at the Pôle d’Expérimentation Avicole de Tours, where the experiment took place (UE PEAT, INRAE, 2018. Experimental Poultry Facility, doi: 10.15454/1.5572326250887292E12). They had access to a wood-chip littered barn (25m^2^) equipped with nesting boxes and perches, and had access to an outside enclosure (approx. 30m^2^) enriched with perches. Water was provided ad libitum, and food was delivered at will once the experiments of each testing day were completed. Birds were kept in a stable social group of 21 individuals on a 6am to 6pm daylight cycle. All experiments took place between 9am and 3pm and the testing order of the individuals was counterbalanced each day.

### Stimuli

**Fig. 1:**
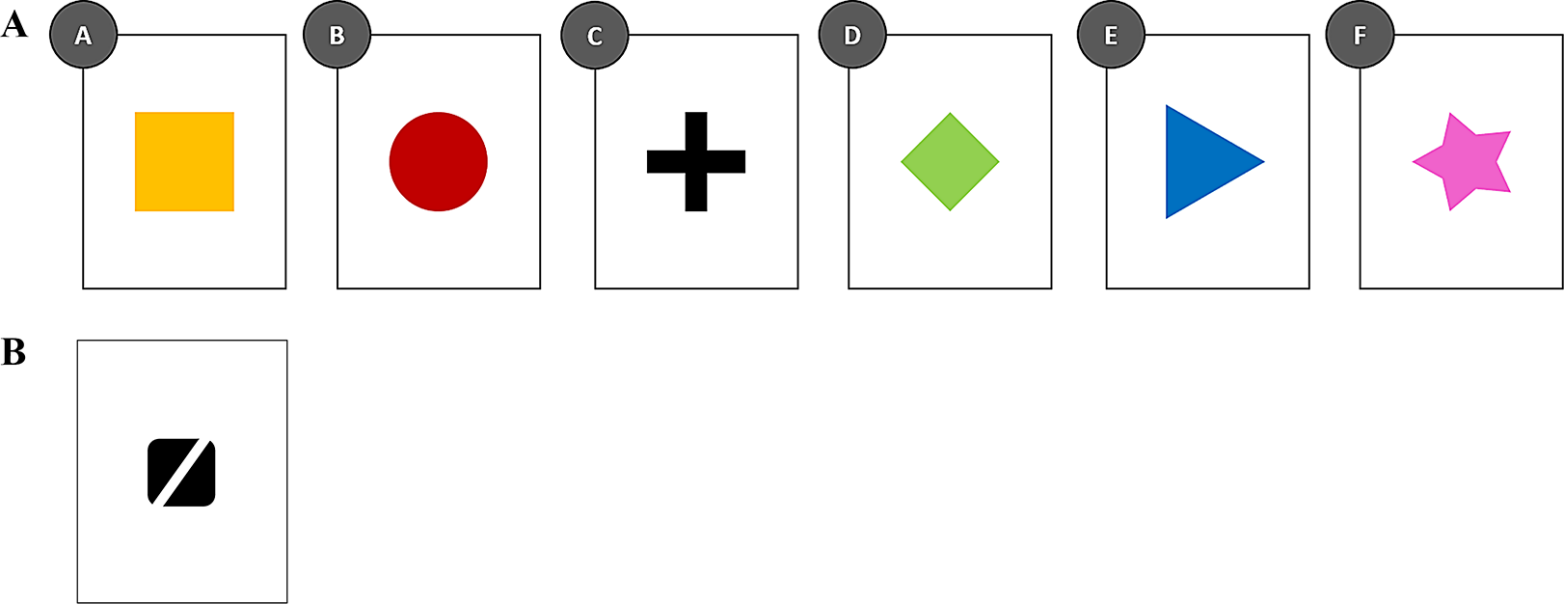
(**A**) Example of the priority sequence of the items for one individual. The colors and shapes correspond to those that have been printed on the cards for the procedure (yellow square, red circle, black cross, green diamond, blue triangle, pink star). The priority order is from item A to item F. (**B**) Illustration of the neural card used to train hens to peck on a card to get the food reward

Each individual was attributed a unique sequence of 6 items, composed of the same items in a different order. The items (4 × 4 cm) were printed in the center of white cards (10 × 8 cm). Each item had a different color (yellow, red, black, green, blue, pink) and a different shape (square, circle, cross, diamond, triangle, star) (Fig. 1a). The colors and the shapes were chosen in order to maximize the difference between the items. With the same purpose, the blank rate (i.e., the percentage of the card that was not covered by perceptual information, here: white parts) and the structural information rate (i.e., the percentage and heterogeneity of the perceptual information of the card) were not controlled. Each individual had a unique sequence order of these items, thus, for clarity, we will name the items depending on their sequence position: A, B, C, D, E and F. Another, neutral card was used to train the hens to peck on cards to get a food reward (Fig. 1b; detailed below).

### Apparatus and general operative

#### Apparatus

**Fig. 2:**
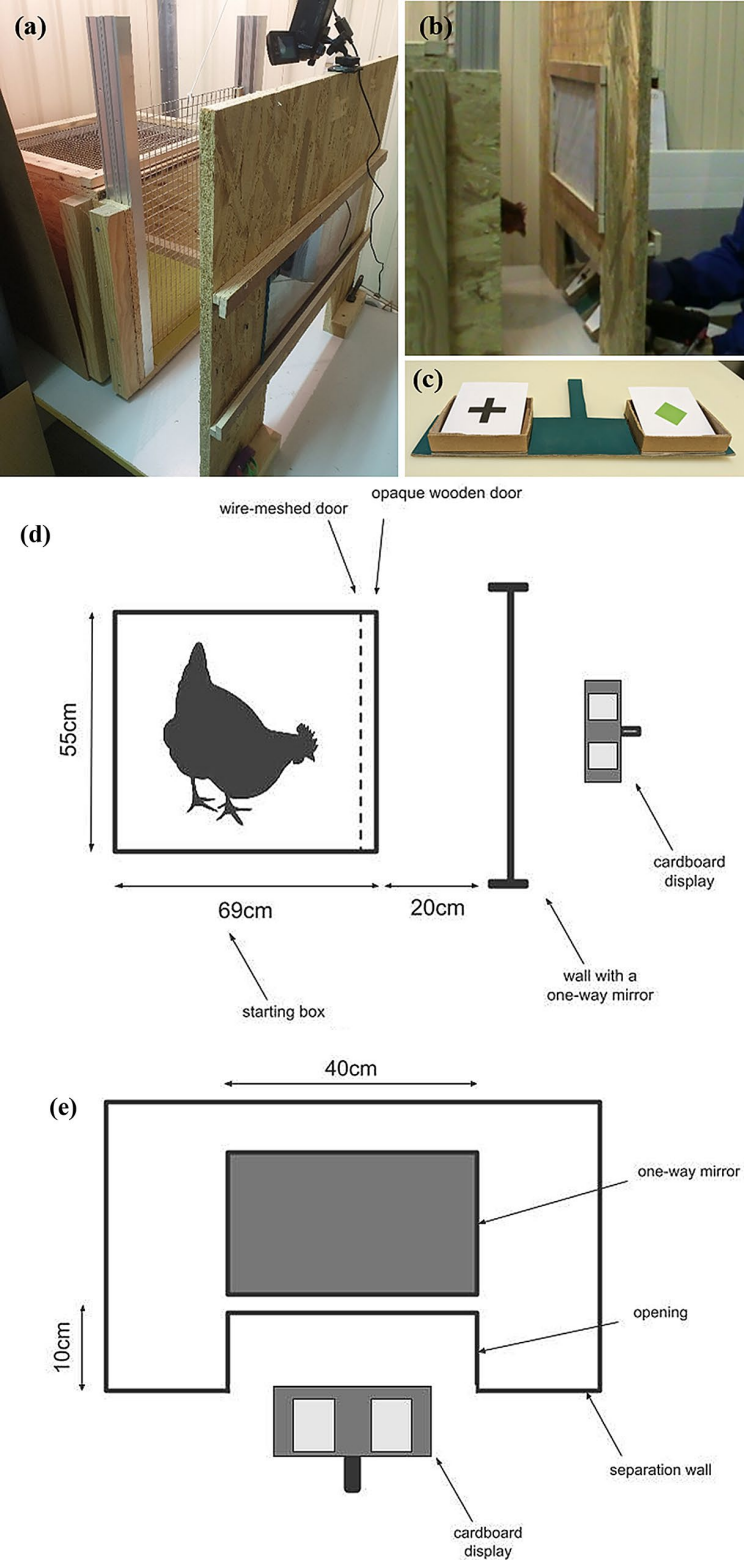
(**a**) Apparatus, with the starting box and the separation wall. (**b**) In each trial, the experimenter showed a pair of cards through the opening on the separation wall, and the tested hen was allowed to choose, by pecking, one of the two items presented. (**c**) The cards were presented on the display. (**d**) Illustration of the top view of the apparatus. (**e**) Illustration of the experimenter’s view of the separation wall between the experimenter and the hen

The apparatus consisted in a starting box and a separation between the box and the experimenter, both made in condensed wood. The starting box (69 cm long x 55 cm large x 80 cm high) was closed with a wire-meshed door (Fig. 2a). A separation wall with a one-way mirror stood 20 cm away from the starting box. This separation wall provided a 10 cm high x 40 cm large opening, allowing the experimenter to present the cards to the hen without her being able to see the experimenter (Fig. 2b). The pairs of cards were presented on a solid cardboard display (Fig. 2c). An illustration of the apparatus is given in Fig. 2d and e.

#### General procedure

Before training, each hen was habituated individually to the new testing environment and to be carried out from the social group and placed in the starting box in a separate, adjacent room. Then, each individual was accustomed to peck on a neutral card (black figure printed on a white card with the same dimensions as the test cards; Fig. 1b) to get mealworms. This habituation was carried out using a clicker-training procedure (Feng et al. 2016).

After this procedure, the stimuli habituation and the training sessions started for each individual. During habituation, the experimenter randomly presented each item of the series, as a prior habituation, and each hen pecked directly on each item regardless of the shapes or the colors. Then, through the training sessions, the hens would learn to choose the correct item from each pair to get the reward: for example, in the series ABC, peck at B when faced with B-C.

For each trial, the pair was prepared out of the sight of the individual. Then, the wire-meshed door was opened and the experimenter presented the pair on the display. A choice was considered when the hen pecked on one card. If her choice was incorrect, the display was immediately taken back and the hen was gently, manually brought back in the box by the experimenter for a 10-second wait. Then, a maximum of 3 correction trials was given with the exact same trial. If her choice was correct, the experimenter clicked and waited for a second peck on the same card to give her the reward (3 mealworms, either for a successful standard trial or a successful correction trial). After each trial, the hen was brought back in the starting box and the inter-trial interval was of 15 to 20 s. If the hen did not respond within 2 min, the experimenter closed the door and waited 10 s before trying again. If the hen did not respond for a total of 3 min, she was considered unmotivated and the session was postponed.

Sessions could last from 20 to 45 min depending on the individual performance (number of correction trials needed: up to 3 per standard trial) and depending on the number of trials in the session. Each individual had one session per day. For each training or testing session, the prior item for each pair was equally presented right and left among the trials (e.g., in the pair CD, the prior item is C), and a prior item was presented no more than twice in a row on the same side. A learning criterion was required at each training step: both in single-pair training and in mixed sessions (detailed below), the hen had to reach a minimum of 75% of success in 2 consecutive sessions with more than 50% of success for each side (right or left) of presentation of the prior item for each pair. However, if an individual reached 90% successful trials or more for both sides within one session, then the learning criterion was considered to be reached. The learning criteria stood to avoid any potential side bias, i.e., systematically pecking on the left or on the right card, independently of the card. In case of a side bias, a maximum of 8 correction sessions were run, that were adapted to the bias and the strength of the bias, on a case-by-case basis.

### Procedure

#### Training sessions

First, during the single-pair training of the premise pairs, each pair of adjacent items was learned in the sequence order (e.g., AB, then BC, etc.). These training sessions comprised 20 trials, with an equal amount of left and right presentations of the prior item. We measured the number of sessions individuals needed to reach the learning criterion for each pair.

**Fig. 3:**
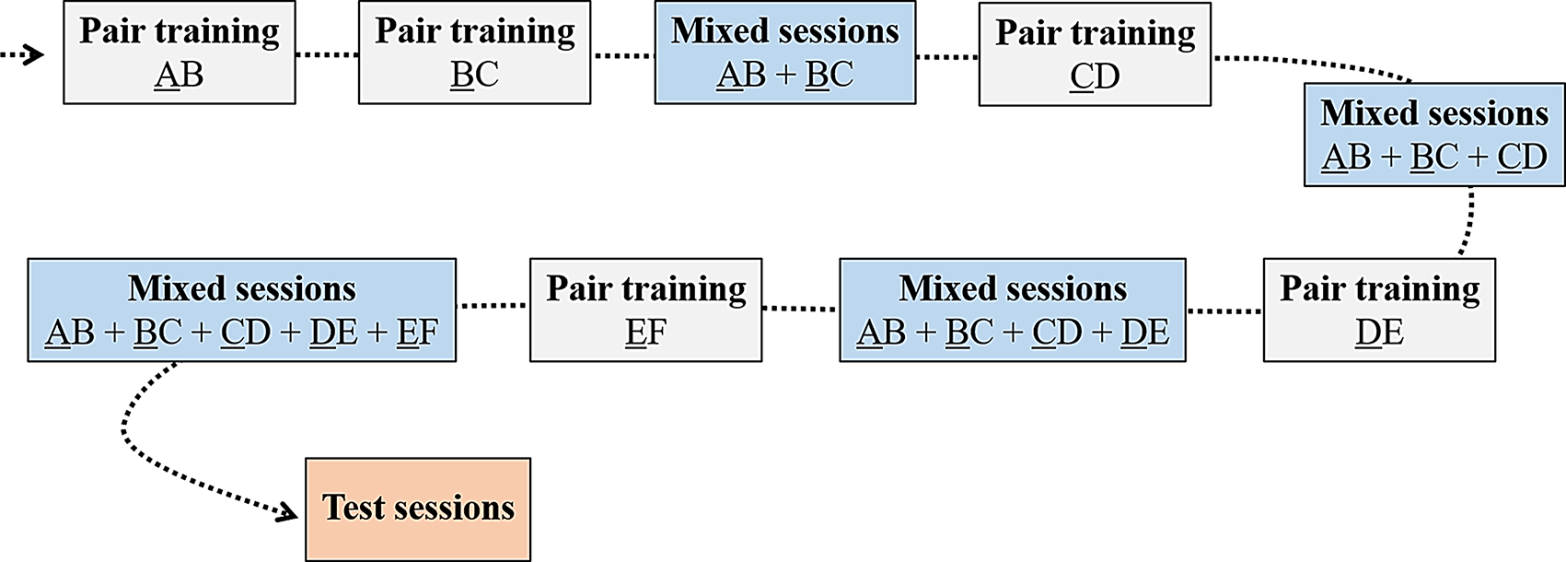
Sequence of the pair training of adjacent items (single-pair training) and of the mixed sessions. Individuals had to pass a learning criterion at each step. The outlined items are the prior items in the pairs

Then, to ensure that hens were able to remember the correct choice for the items that were involved in two different situations (e.g., C is the prior item the hen had to peck on in CD but not in BC), mixed sessions, where all pairs previously learned were retrained, were added to our training schedule. These mixed sessions were included after the training of BC, CD, DE and EF, respectively (Fig. 3). This hybrid training procedure (successive pair training intermixed with mixed sessions) has been shown to lead to a faster significant performance for the premise pairs (Daniels et al. 2014). For a consistency of the number of trials per pair per session, and considering the conditions of prior items presentations, we allocated a total number of trials per session accordingly. Mixed sessions after BC training sessions had a total of 20 trials; those after CD had 18 trials; those after DE had 32 trials; those after EF had 40 trials. The number of presentations of each pair was equal in each session. We controlled afterwards that the number of presentations of each pair did not influence the performance in inference trials (Supplementary Materials S2, § 1).

We measured the number of sessions the individuals needed to reach the learning criterion for each mixed step. For the last three mixed sessions (which included the 5 premise pairs), a baseline performance (percentage of success) and the response latency were measured for each premise pair.

#### Test sessions

Each individual had 12 test sessions with 46 trials each: 40 trials of premise pairs (8 trials per pair), 3 control trials and 3 inference trials. Control and inference trials were non-rewarded and with no correction trials.

Extremity items were not used in inference trials as they are either always reinforced (A) or never reinforced (F). Thus, pairs of nonadjacent items involving an extremity item (e.g., *A*C or C*F*) do not test for TI but can be used to demonstrate the basic understanding of associative values according to the reinforcement of each item during training (end-anchor effect, von Fersen et al. 1991). Control trials consisted in pairs of nonadjacent items with either the very first item A or the very last item F (AC, AD, AE, BF, CF or DF). These A and F control trials were pseudo-randomly distributed and equally presented among the 12 test sessions.

Inference trials were trials of nonadjacent items (but not extremity items) for which transitive inference was needed to give the correct response. The pairs involved items that were both equally reinforced (for example, in BD: B + C- and D + E-) and non-reinforced (for example, in BD: A + B- and C + D-) during the training. The six-term series enabled to test TI through three inference trials (BD, BE and CE). Each one of the possible inference trials was presented once per test session. The sides of presentation of the prior item for each possible inference trial, were pseudo-randomized and equalized within two sessions.

During the test sessions, the performance for the premise pairs was tracked. In case of a side- or an item-bias, we integrated optional correction sessions to ensure an equalized level of performance for every premise pair throughout test sessions. These sessions contained at least the problematic pair and its two adjacent pairs, with a minimum of 12 trials per session, with 50 to 75% of the trials being allocated to fix the issue (i.e., a higher proportion of the problematic item(s), or a higher proportion of prior items on one side or the other). A maximum of two correction sessions was added each two test sessions.

We measured the number of successful trials for the premise pair trials, for control trials, and for inference trials. We measured the response latency for control trials and for inference trials.

### Statistical analysis

All statistical analyses were performed using R version 4.2.1 (R Core Team 2022). We considered p-values below 0.05 to be significant, and p-values below 0.1 as a statistical tendency, for all statistical analyses. Chance level was considered at 50% of success. A non-parametric approach was preferred due to the small sample size (6 to 4 individuals).

The response variables were either the number of sessions to criteria, the percentage of success, or the response latency. The fixed variables were the side for the presentation of the prior item, the trial type, the pair and the number of the session. Anova permutational models were performed when several explanatory variables were to be considered, with individuals as random effect (aovperm function, package *permuco*, Frossard and Renaud 2021; np = 10000, type = permutation). Symmetry tests or one-way permutation tests were run to analyze the effect of a fixed variable with more than 2 modalities on the response, when the data were paired or not, respectively (package *coin*, Hothorn et al. 2006; two-tailed comparisons, np = 10000). Pairwise permutation t-tests were run for Posthoc analyses to detail the comparison between more than two modalities (pairwise.perm.t.test function, package *RVAideMemoire*, Hervé 2022; np = 10000, Holm correction). Two-tailed Wilcoxon rank-sum tests were used to assess the statistical significance of a comparison against the chance level, with Holm correction when appropriate (manual correction). Two-tailed exact binomial tests were used to test the statistical significance of individual performances (binom.test function). Homogeneity of variances was assessed with Levene tests before model fitting and before running multiple comparison analyses. Graphics were performed with the package ggplot2 (Wickham et al. 2019).

For statistical analyses including less than 10 responses, the results are reported as the median and the first and third quartiles (MED [Q1:Q3]). For other statistical analyses, the results are reported as the mean and the standard deviation (MEAN +/- SD).

As some training and testing parameters could have caused the use of associative cues in test trials, we ran supplementary analyses afterwards to control for some eventual response biases because of the sessions’ configuration (Guez and Audley 2013). We controlled for an effect of the reinforcement history and the reinforcement ratio of the items during training (Daisley et al. 2010, 2021; Hotta et al. 2020; Okouchi and Lattal 2006; Lazareva et al. 2004) and of the configuration of premise pair trials which were presented just before the test trials in test sessions (Russell et al. 1996). These analyses are detailed in Supplementary Materials S1.

## Results

### Training sessions

The details of the number of sessions to reach the learning criterion at each step for each individual are presented in Supplementary Materials S1, Table S1. Overall, only a few sessions have been postponed due to lack of motivation (premise pairs training stage: Starr = 1; mixed pairs training stage: Starr = 2, Daenerys = 1, Savana = 1; testing stage: Savana = 2, Octo = 1).

#### Single-pair training of the premise pairs (i.e., pairs of successive items)

At the group level, the mean number of sessions needed to reach the learning criteria did not differ between the different pairs (variance homogeneity, one-way permutation test, chi2 = 4.5469, df = 4, *p* = 0.337; global median number of sessions = 3 [3:4] sessions to reach the criterion). The range of correction trials needed during this stage went from 0 to 44 correction trials per session, with strong variations depending on the individual and on the training progress (mean number per session at the group level: 9.33 ± 10.65 correction trials; Supplementary Materials, Table S2).

#### Mixed sessions

At the group level, the mean number of sessions needed to reach the learning criterion was significantly different depending on the number of pairs included in the session (no variance homogeneity, one-way permutation test, chi2 = 12.203, df = 3, *p* = 0.007). More precisely, the learning speed was higher in the mixed sessions including pairs [AB to BC] compared to other stages of mixed sessions (significant with pairs [AB to CD]: *p* = 0.046; statistical tendency with [AB to DE]: *p* = 0.082 and with [AB to EF]: *p* = 0.074; pairwise permutation t-test). The range of correction trials needed during this training went from 0 to 22 correction trials per session, with strong variations depending on the individual (mean number per session at the group level: 9.04 ± 2.94 correction trials; Supplementary Materials, Table S2). The mean number of correction sessions needed during this stage was 22.50 ± 5.97 sessions (Daenerys: 31, Dion: 21, Octo: 21, Starr: 17).

#### Baseline performance for the premise pairs

**Fig. 4:**
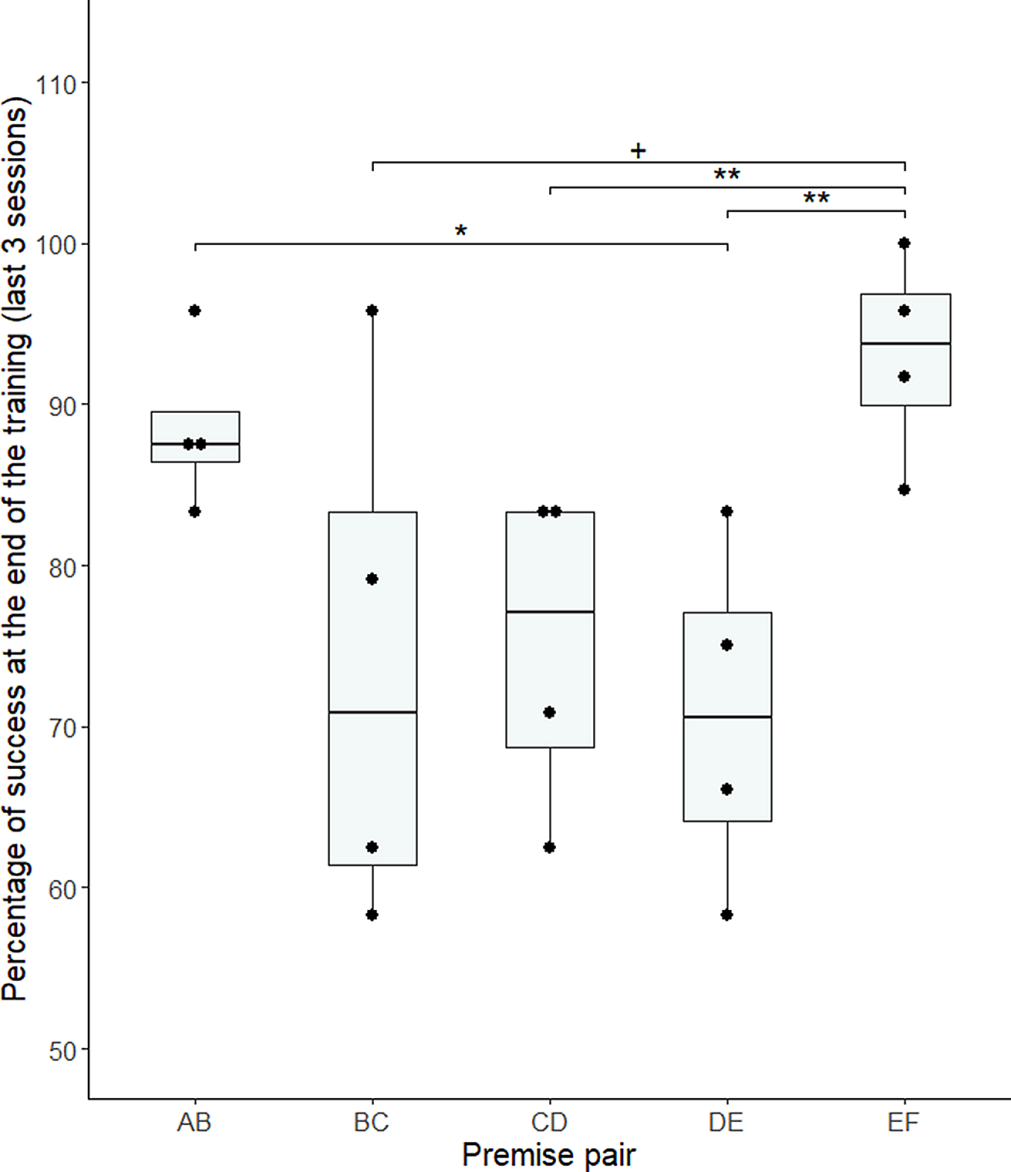
Percentage of success at the group level for each premise pair at the end of the training (3 last mixed sessions – 8 trials per pair per session), for the four individuals that passed all the learning criteria. *: *p* < 0.05; **: *p* < 0.01; +: *p* < 0.1

**Fig. 5:**
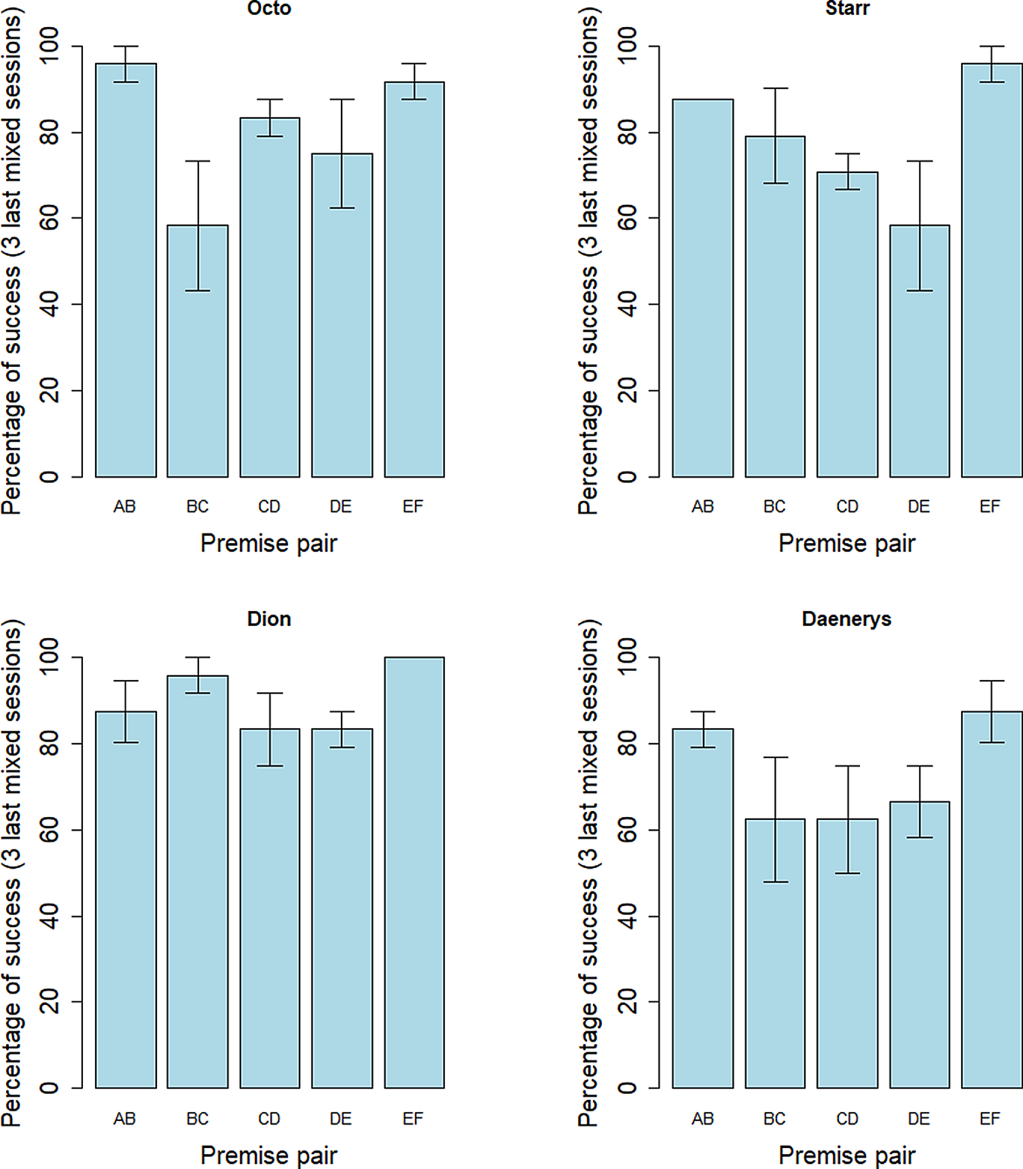
Mean percentage of success at the individual level for each premise pair at the end of the training (3 last mixed sessions), for the four individuals that passed all the learning criteria. At the individual level, there was no significant difference in the performance of the premise pairs

Four out of six individuals successfully finished the training by reaching the learning criteria (Supplementary Materials, Table S3). Two individuals (Savana and Elizabeth) have not progressed to the required steps and were not included in the following analyses. In the last 3 mixed sessions, the mean performance for the premise pairs was of 80.42 +/- 17.88% of success, which is higher than chance level (two-tailed Wilcoxon rank-sum test, V = 1526.5, *p* < 0.001; Supplementary Materials, Table S4). We used a permutational anova model analysis to estimate the effect of the pair presented and of the side of the prior item, on the percentage of success. We found an effect of the side (F = 8.69, *p* = 0.011), with a side bias for left over right at the group level (mean performance when the prior item is on the left = 85.69 +/- 19.29%; on the right = 74.81 +/- 25.59%). This side bias was not different between the pairs (F = 1.464, *p* = 0.260). Moreover, we found an effect of the pair presented on the performance (F = 3.343, *p* = 0.038; Fig. 4). A multiple pairwise analysis revealed significant performance differences between pairs AB and DE (*p* = 0.035), CD and EF (*p* = 0.007) and DE and EF (*p* = 0.002) and a statistical tendency for the difference between pairs BC and EF (*p* = 0.053; pairwise permutation t-test). The performance for the premise pairs at the end of training for each individual is detailed in Fig. 5.

#### Response latency for the premise pairs in the 3 last mixed sessions

**Fig. 6:**
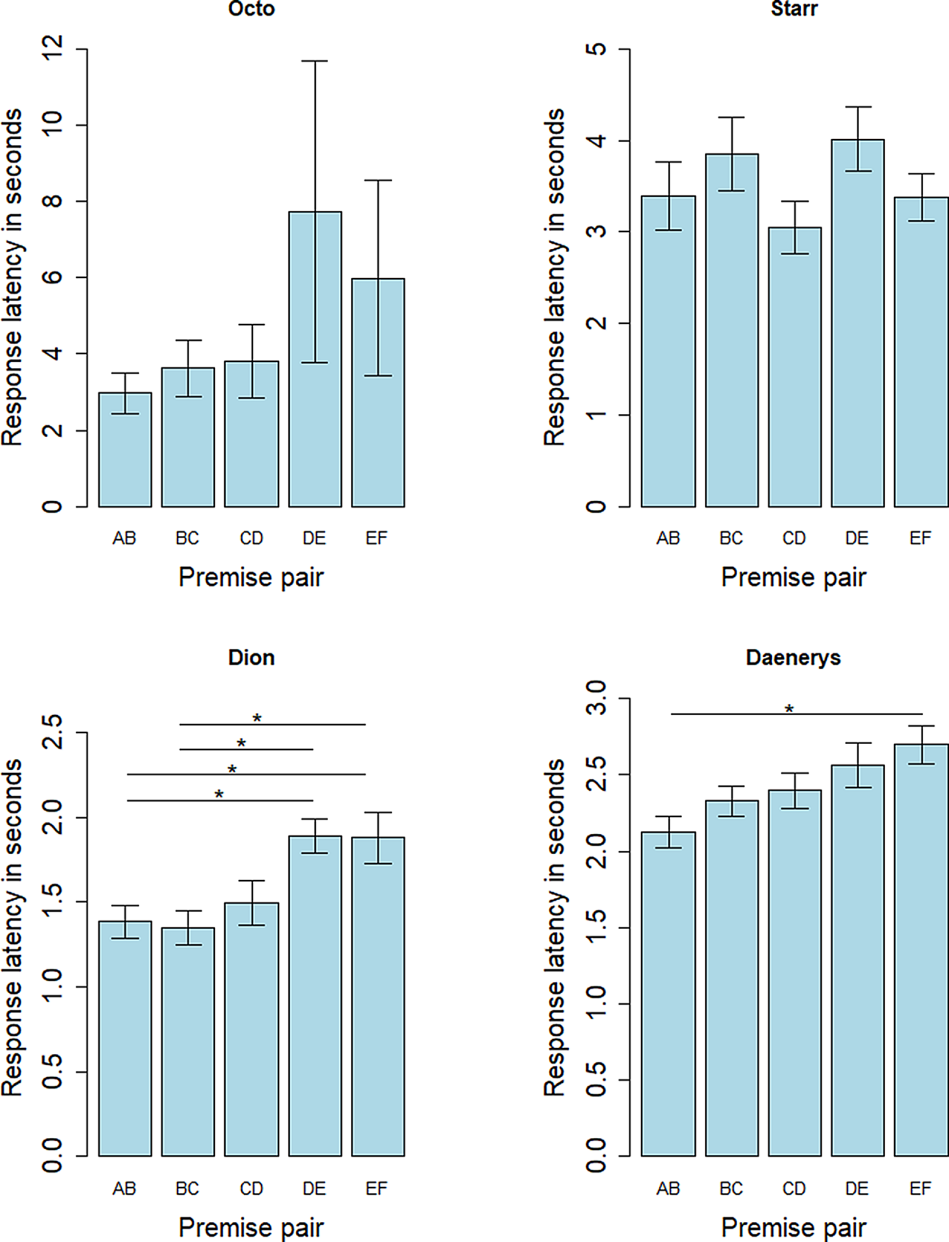
Mean response latency in seconds for each premise pair for each individual, at the end of the training (3 last mixed sessions). *: *p* < 0.05 (pairwise permutation t-test)

The mean response latency for the premise pairs was 3.097 +/- 3.335 s. We found no difference in the response latency between the premise pairs (one-way permutation test, variance homogeneity; chi2 = 4.9606, df = 4, *p* = 0.291). At the individual level, there was a significant difference in the response latency between the pairs for two individuals, with a slower response latency for DE and for EF (for Dion: between AB and DE: *p* = 0.020; between BC and DE: *p* = 0.014; between AB and EF: *p* = 0.048; between BC and EF: *p* = 0.048; for Daenerys: between AB and EF, *p* = 0.018; pairwise permutation t-test). The response latency for each premise pair at the end of the training for each individual is detailed in Fig. 6.

### Test sessions

Four individuals successfully finished the training and were tested for transitive inference. Their performance in control trials validated the basic understanding of the associative rule of the (non-)reinforced extremity items, as each hen performed better than chance level (total of 36 control trials; group median performance = 98.61 [95.14:100] %; binomial tests, *p* < 0.05; performance for A- trials = 97.22 [94.44:100] %; for F- trials = 100 [95.83:100] %). During the testing stage, Octo got 4 out of 8 possible correction sessions between the test sessions, Daenerys and Starr got 5, and Dion got 6.

#### TI performance

All hens performed better than the chance level (total in 36 inference trials; 80.56% for Octo, 83.33% for Starr, 94.44% for Dion and 72.22% for Daenerys; binomial tests, *p* < 0.05). The median performance at the group level was 81.94%. Starr, Dion and Daenerys were successful in their very first inference trial. Within the 12 first inference trials, Dion and Daenerys performed significantly better than the chance level (12/12 and 10/12, respectively; binomial tests, *p* < 0.05; 7/12 for Octo and Starr). We found no side bias in test trials (anova permutation model analysis; F = 1.19, *p* = 0.314), either in control or in inference trials (no interaction; F = 0.629, *p* = 0.432).

#### Comparison of the different inference trials

**Fig. 7:**
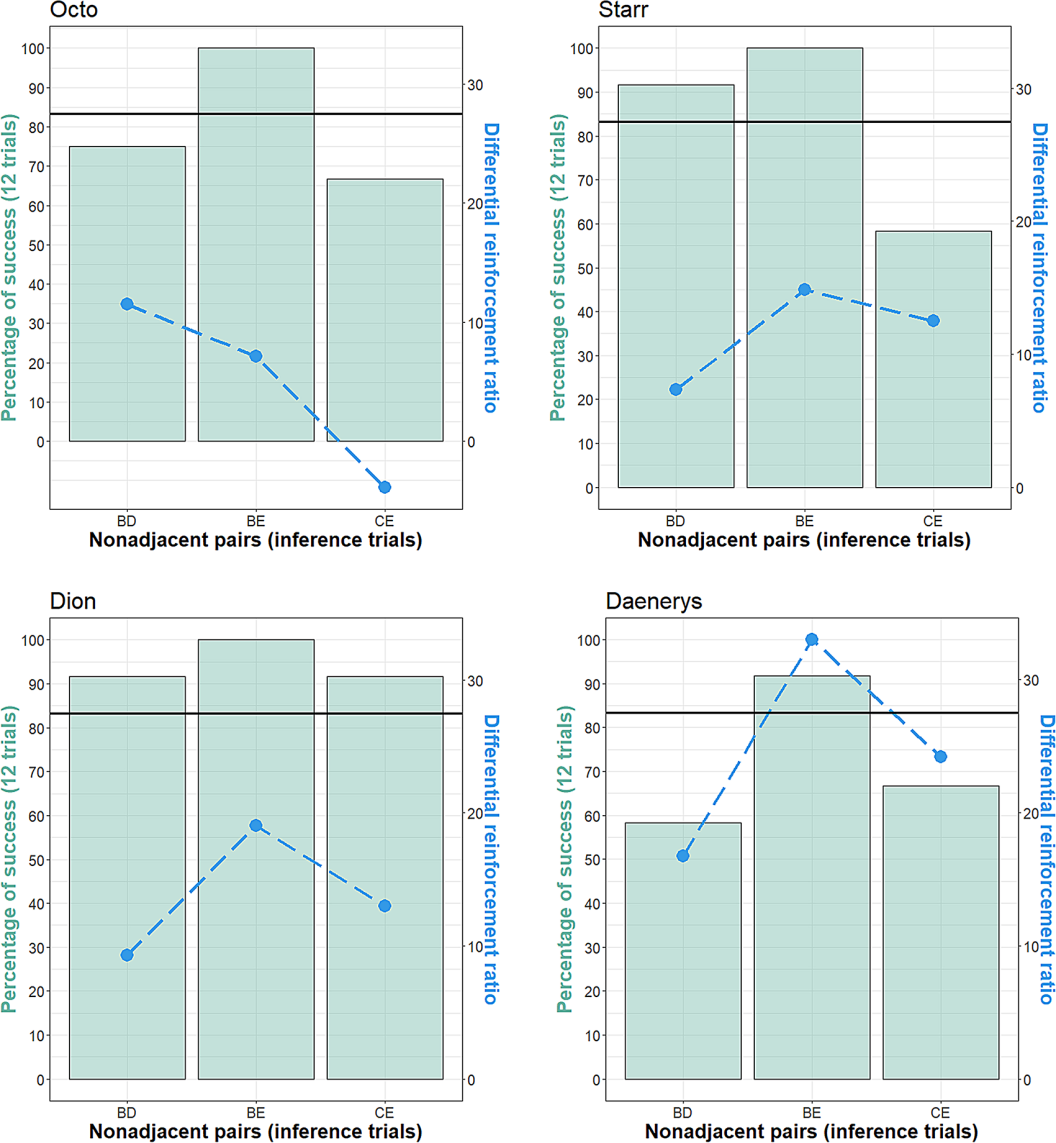
Detailed performance for each type of inference trial for each individual. Left y-axis: percentage of success in 12 inference trials; the black horizontal line indicates the performance to reach to be significantly higher than the chance level, i.e., 83,33% (10 trials out of 12). Right y-axis: differential ratio of reinforcement in percentage (see Supplementary Materials S2); for example, the higher D_BD_ is, the more B has been reinforced in comparison to D

At the group level, we found a statistical tendency for the difference in the performance between the 3 trial types at the group level, with a higher performance for BE trials compared to BD and CE trials (variance homogeneity, symmetry test, maxT = 2.250, *p* = 0.063; median performance for BE = 100 [97.9:100] %; for BD = 83.30 [70.83:91.60] %; for CE = 66 [64.08:70.47] %). At the individual level, each hen performed better than 50% of success for each trial type (BD, BE and CE). All individuals performed significantly better than chance level when tested with BE, two individuals performed significantly better than chance level when tested with BD, and one individual when tested with CE (statistical significance for more than 10 successful trials among 12 for each inference trial possibility; two-tailed binomial test, *p* < 0.05; Fig. 7). The performance in inference trials could not be explained by the differential ratio reinforcement of the items during training, except for Daenerys (Fig. 7, Supplementary Material S2, § 1).

#### Response latency

We found a significant difference in the response latency between premise pairs, control trials and inference trials (One-way permutation test, chi2 = 7.553, df = 2, *p* = 0.023) with a significant difference between premise pairs and control trials (pairwise permutation t-test, *p* < 0.001; mean response latency for premises pairs trials = 3.0965 +/-3.33 s, for control trials = 2.043 +/-1.106 s, for inference trials = 2.519 +/- 2.189 s). We found no significant difference in the response latency between the different pairs presented in control and in inference trials (homogeneity of variances, One-way permutation test, chi2 = 7.89, df = 8, *p* = 0.444). In inference trials, the mean response latency for BD trials was of 2.271 +/- 1.201 s; for BE trials = 2.308 +/- 1.207 s; and for CE trials = 2.933 +/- 3.438 s.

### Control for confounding factors on choice behavior and TI performance

We analyzed whether the hens could have relied on other cues to be successful in test trials. These analyses are detailed in the Supplementary Materials S2. The results show that the reinforcement ratios of the items and the configuration of the test sessions could not have allowed most hens to perform higher than the chance level in test trials, as (1) neither the reinforcement history nor the reinforcement ratio of the items resulting from the training stage influenced the performance in test trials, excepted for Daenerys, (2) nor the configuration of the previous pair trial influenced the choice behavior in the following test trial.

Interestingly, the fact that test trials were not rewarded might have affected the performance of the hens in premise pairs during test sessions. A permutation anova analysis showed that the choice to peck at an item in a premise pair significantly increased when the individual had pecked on this item in the previous non-rewarded test trial (control or inference trial) but only if it was presented on the other side (anova permutation model analysis; interaction: F = 7.108, *p* = 0.008). In parallel, whether hens gave the correct (C_T_) or the incorrect (I_T_) response at test trials did not significantly impact their performance (correct C_H_ or incorrect I_H_) at the next premise pair trial which presented one of the same items (two-tailed Wilcoxon tests with Holm correction; mean occurrence of I_T_I_H_=0, I_T_C_H_=10 +/- 4.97, C_T_I_H_=0, C_T_C_H_=42.5 +/- 9.85).

## Discussion

Our results suggest that the hens were capable of transitive inference when confronted with the 6-term series task. This confirms what has been observed in previous studies in poultry, in which a set up with a five-item series had been used (in chicks: Daisley et al. 2010, 2021; in geese: Weiß et al. 2010). By performing deeper analyses, we found an inter-individual variability in the resolution of the task. The TI performance of most hens was not impacted neither by the reinforcement ratios of the items, nor by the configuration of the sessions, supporting the use of transitive inference in this task, and making it harder to support a purely associative-based resolution of the task (except for one individual, Daenerys). Notably, one hen showed a very high performance for all premise pairs and for the three different inference trials, and her performance didn’t seem to be impacted by the common associative effects (detailed below). Overall, the present study expands our knowledge on how chickens learn and solve a relational task.

### Learning performance

Overall, the hens needed a mean of 51.25 +/- 13.43 sessions to complete the training stage. Hens were faster to learn the six-term series than greylag geese confronted with a 5-term series with a similar hybrid training procedure (mean of 83.4 +/-17.1 sessions; Weiß et al. 2010). Two main reasons might explain this difference. Firstly, the learning criteria may have been more demanding in Weiß et al.’s study as they included a criterion of 81.25% of success over the 16 last consecutive trials for each premise pair. Secondly, our hens were tested in a controlled environment without further distractions, which was not the case for the greylag geese.

As in greylag geese (Weiß et al. 2010) and in pigeons (Clement and Zentall 2003), the hens learned the first two premise pairs at a similar speed (Supplementary material S2, Table S3). While these authors considered this performance to be part of a natural tendency of the animals to choose a familiar stimulus over a novel one, we observed a different pattern. We observed that the hens tended to apply the rule previously learned at the start of the training sessions for each premise pair (5 to 10 first trials), that is, they tended to first avoid the item they had not to peck at in the previous pair (e.g., do not peck at B in BC because that was the item to not peck at in AB). A difficulty to step to a new pair has already been found in other species (Treichler and Van Tilburg 1996 in macaques; Bond et al. 2003 in corvids). A possible explanation could be that hens tended to apply a previous rule when facing new situations, which could be referenced as rule generalization. Another explanation could be that the hens demonstrated a choice based on a reject-controlling relation, as they tended to conduct their choice based on the non-rewarded item S-, by rejecting it when the previous rewarded item S + was replaced by another item (McIlvane and Dube 1992; Goulart et al. 2015). Both hypotheses presuppose that the learning speed of the new pair could rather be attributed to a high behavioral flexibility to learn the new rule (see Degrande et al. 2022).

The comparison of the performance between the premise pairs has been shown to bring further information about the cognitive process that could be at stake in the n-term series task (Bond et al. 2010). When comparing the performance between the premise pairs at the end of the training, each hen showed a better performance for AB and EF compared to the other pairs. This performance can be associated with what is known as the end-anchor effect. In simple terms, this serial position effect (i.e., a performance that depends on the serial position of the items in the ordinal series) implies better performance when the pair includes an extremity item (here, A or F; Allen 2006). This effect can be explained through the strong conditioning (positive and negative, respectively) that comes with the very first item of the series, that is always reinforced, and the very last, that is never reinforced.

Concerning the response latency, two out of four individuals showed a choice speed that was dependent on the serial position of the pairs, with a slower response for the pairs at a greater distance from A. Several hypotheses report this result, and notably, the hypothesis of a mental representation of the series and, on the opposite, the hypothesis of a performance based on the associative, rewarding value of the items. Some authors argue that this effect suggests a mental representation of the ordinal series (Terrace 2005): the item A being the reference point of the spatial representation of the series, the response latency for the premise pairs increases with the distance from A. This result was significant for Daenerys (AB < EF) and for Dion (AB < DE and EF, and BC < DE and EF). However, associative hypotheses can also account for this effect. For example, the *first-item effect* hypothesis (von Fersen et al. 1991) claims that the first item of the series (A) has a strong positive value and is thus associated with a shorter response latency, and that the response latency for the premise pairs involving the following items in the series will decrease gradually with the distance to A. Another hypothesis is that a higher number of presentations of the pairs during training might generate a shorter response latency for these pairs. The pattern of response latency for the premise pairs needs to be further investigated as the result is not significant at the group level in our study.

At the individual level, two out of the six individuals did not manage to end the training stage, by not being able to mix the first two premise pairs (Elizabeth) or not being able to mix the five premise pairs together (Savana). The fact that some individuals never reach the learning criteria has been found in other species (e.g., in pigeons: von Fersen et al. 1991). The n-term task, commonly used for TI testing, involves some individual parameters that are prerequisites to the task and that can strongly impact the performance, independent of the ability to perform TI. Independently of inferential and relational memory abilities, this task requires a long-term memory, a retrieval capacity, a certain level of behavioral flexibility, and the ability to apply a different rule for the same item depending on the context of presentation (i.e., depending on the pair). Personality and social structure issues have been shown to impact these parameters in birds (chicks: Daisley et al. 2021; geese: Weiß et al. 2010). This observation calls for precautions when testing for TI in animals with the common n-term series task.

Finally, we found a significant side bias for left over right in the last mixed sessions of the relational training. In the same way, Daisley et al. (2010) found that chicks who could use their left eye only had a better TI performance in comparison to chicks who could use their right eye only. As the birds’ visual pathway has been shown to be contralateral (Deng and Rogers 1998), the authors conclude that the right hemisphere may be more implicated in TI, which is consistent with a development of TI capacities through relational representations in mammals (see for example Dusek and Eichenbaum 1997). However, we did not find a significant side bias in the TI trials, which could mean that the cognitive process implied in relational learning is different than the one implied in relational retrieval in birds. Lastly, we cannot rule out the possibility that this side bias has been caused by another, internal (individual life history) or external (apparatus design details) cause that we had no control over, and that we couldn’t reveal the same side bias in test trials due to a smaller number of trials.

### Transitive inference performance

Hens showed transitive inference in the 6-term series task. Each hen performed significantly better than chance in 36 inference trials (non-rewarded test trials), and two hens did it in 12 trials. Notably, one individual (Dion) performed significantly better than chance level for each of the three different trial types (BD, BE and CE). Because our study controlled for several alternative explanations, such as a strategy based on the reinforcement ratio of the items or a possible performance based on the configuration of the sessions (see Supplementary Materials S2), it is likely that the hens were using transitive inference as the strategy for completing the task. For example, TI performance was not dependent on the reinforcement ratio of the items for three out of the four hens, which is an argument against a performance relying solely on associative responses.

The six-term series enabled us to study further the cognitive resolution of the task through the three inference trial types. Hens performed better in inference trials with more distant items, i.e., in the nonadjacent pair BE (performance significantly better than chance for the four hens), compared to pairs BD and CE (significantly better than chance for two hens and for one hen, respectively). Such a performance, that is related to the distance between two items, refers to different hypotheses depending on the authors. Within the linear representation theory, it is referred to as the symbolic distance effect that supposes that the individual made a mental linear representation of the series to make a choice (Moyers et al. 2018). It has been found in other species as pigeons (von Fersen et al. 1991; Daniels et al. 2014), corvids (Bond et al. 2003) or even in humans (for example: Bryant and Trabasso 1971) and is consistent with linear models of TI (von Fersen et al. 1991; Daniels et al. 2014). However, this effect could also have been caused by side effects of the training procedure, like the fact that the premise pairs were trained in a temporal order that was consistent with their ordinal rank, or the delay from the initial presentation of the pair to the start of the test sessions. Thus, we cannot state from our results whether the hens mentally organized the items of the series along a linear representation or not (Couvillon and Bitterman 1992; MacLean et al. 2008).

In parallel, this symbolic distance effect is also explainable by the Value Transfer Theory (VTT) from von Fersen et al. (1991) or derived associative-based models (see for example Zentall et al. 1996; discussed in: Allen 2006; Vasconcelos 2008; Guez and Audley 2013). In these models, each item is being transferred a partial associative value from its adjacent items in the series, depending on the reward contingency that occurs in the pairs presented. This way, A transfers its positive value along its adjacent items, and F transfers its non-rewarding, negative value along its adjacent items. This transfer mechanism could thus make it easier to respond to BE through associative values than BD or CE.

A last concern about this issue is that, according to the symbolic distance between the items, the performance for the pairs BD and CE should have been similar. However, we found that the performance for BD was better than that for CE for two out of four individuals (not significant). The decreasing TI performance from BE to BD, and from BD to CE has also been found in pigeons in Daniels et al. (2014) at the group level. Overall, it is possible that the worse performance in CE is related to a recency effect for the learning of the pair EF (with E rewarded), which hypothesis must be validated through further studies (Bond et al. 2003).

Focusing on the performance of each individual separately, we show that the hens might have used different cognitive strategies to solve the TI task. At one extreme, Dion probably solved the task through a relational representation of the series: she showed a high performance for the premise pairs and showed no first- or last-item effect, and an almost perfect performance for the three different inference trials. Her performance highlights the relevance of studying the response latency to observe potential response differences between the pairs at a high level of performance. At the other extreme, Daenerys may have used a more associative strategy, as her performance could be predicted by the values of the differential ratio of reinforcement at the end of her training. Her learning performances and her inference performance seem to match an associative model including a transfer of associative values through the items.

More broadly, the relational learning involved in this task might be encoded through multiple cognitive processes. The TI task, as it is designed in most experiments, might simultaneously involve both cognitive- (through a relational representation) and associative- (through associative-based models) strategies (Jacobs 2006; Lazareva and Wasserman 2006; Bond et al. 2010). The current state of the art suggests that inferential cognitive processes and associative processes act in a coupled manner in the context of relational inference capacities (Bond et al. 2010; Jacobs 2006; Lazareva and Wasserman 2006; Völter and Call 2017; Eichenbaum and Cohen 2014; Whittington et al. 2020). This statement gives rise to the notion of multi-action for the resolution systems that are involved in such capacity (Moors 2014), making it possible to speak of a continuous scale within the associative-inferential dichotomy (Petty 2006). Finally, as we cannot clearly differentiate the cognitive processes at stake from our results, future research deepening these analyses, and carried out with larger sample sizes, are needed to shed light on the cognitive processes at stake in individual chickens in this TI task.

## Conclusion and perspectives

To our knowledge, this is the first attempt to further study the underlying process of transitive inference in the domestic chicken, and more generally in *Galloanserae*. Our results confirm that adult chickens are capable of transitive inference. Moreover, we managed to collect relevant additional information from the learning stage, about the response latency to the different trial types, and to control for some alternative cognitive hypotheses. This experiment enabled us to deepen our knowledge of the possible processes involved for this transitive task in chickens.

Overall, our results suggest that the transitive response of some individuals cannot be explained just in terms of an associative-based model and is more likely to demonstrate a relational inference capacity, suggesting a mental representation of the series. Since our results resemble those found in *Neoaves* (pigeons and corvids), our hypothesis is that TI is based on the same cognitive processes in both phylogenetic groups, and that the cognitive strategy to solve the transitive task might be driven mainly by individual parameters within species. Our study brings additional information about the cognitive capacities in chickens, which might be more complex than often assumed.

## Electronic supplementary material

Below is the link to the electronic supplementary material.

Supplementary Material 1

## Data Availability

The data that support the study findings is hosted in the INRAE data repository at 10.57745/TOXZ8L and is available on reasonable request from the corresponding author at rachel.degrande@gmail.com.
