# Supplementary material for: Transitive reasoning in the adult domestic hen in a six-term series task
Source: Anim Cogn. 2024 Nov 19;27(1):77. doi: 10.1007/s10071-024-01914-1 (PMC11576640; doi:10.1007/s10071-024-01914-1)
Supplement: Supplementary file 1 — Supplementary Material 1 [file 10071_2024_1914_MOESM1_ESM.pdf]

SUPPLEMENTARY MATERIALS

Transitive reasoning in the adult domestic hen in a six-term series task

Degrande R. [a], Amichaud O.[c], Piégu B. [a], Cornilleau F.[a], Jardat P. [a], Ferreira V.H.B. [a], Colson V.[b], Lansade L. [a], Calandreau L.[a]

[a] CNRS, INRAE, Université de Tours, PRC (Physiologie de la Reproduction et des Comportements), F-37380, Nouzilly, Indre-et-Loire, France

[b] Laboratoire de Physiologie et Génomique des Poissons, INRAE, Rennes, France

[c] IFIP - Institut du Porc, 9 Boulevard du Trieux, 35740 Pacé, France

Corresponding author: rachel.degrande@gmail.com

Supplementary Material S1: Details about the training and testing performances

Supplementary Table S1. Number of sessions each individual (columns) needed to reach the learning criterion for each training step (lines). NC: the individual did not reach the learning criterion in the constraints imposed and is thus removed from the experiment.

	Savana	Elizabeth	Octo	Starr	Dion	Daenerys	Mean
AB	4	4	3	3	3	2	3.17
BC	4	3	3	2	4	4	3.33
CD	5		3	3	3	3	3.40
DE	3		4	3	2	3	3.00
EF	5		9	3	3	3	4.60
Mixed sessions with AB to BC	2	NC	2	2	3	3	2.40
Mixed sessions with AB to CD	9		10	9	6	7	8.20
Mixed sessions with AB to DE	11		3	4	9	13	8.00
Mixed sessions with AB to EF	NC		11	10	10	16	11.75
Total sessions to end the training			69	39	43	54	51,25

<u>Supplementary Table S2. Detail of the number of correction trials needed during training for each individual.</u> (a) Mean and standard deviation for the number of correction trials needed per individual depending on the training stage. (b) Mean number and standard deviation for the number of correction trials needed per individual during the premise pair and during the mixed training stages.

(a)

Training stage	Individual	Mean number of correction trial per session	Standard deviation
couple_1	Daenerys	5,00	1,41
	Dion	9,67	13,32
	Octo	11,67	15,14
	Starr	8,00	12,17
couple_2	Daenerys	9,00	9,20
	Dion	9,50	10,97
	Octo	4,33	6,66
	Starr	5,50	0,71
couple_3	Daenerys	4,33	5,13
	Dion	7,67	12,42
	Octo	13,33	15,37
	Starr	11,00	18,19
couple_4	Daenerys	11,67	15,14
	Dion	22,00	31,11
	Octo	5,50	1,91
	Starr	6,67	8,14
couple_5	Daenerys	11,00	16,46
	Dion	16,67	17,01
	Octo	9,33	6,20
	Starr	7,67	9,07
mixed couples 1 to 2	Daenerys	13	7,81
	Dion	5,33	4,93
	Octo	2	1,41
	Starr	0,5	0,71
mixed couples 1 to 3	Daenerys	10,4	2,19
	Dion	7,2	5,5
	Octo	11,6	4,04
	Starr	9	4

mixed couples 1 to 4	Daenerys	13,6	2,07
	Dion	4,75	2,22
	Octo	6,5	0,71
	Starr	5	1,41
mixed couples 1 to 5	Daenerys	16,33	2,58
	Dion	11,75	8,14
	Octo	14	5,74
	Starr	13,6	3,36

(b)

Training stage	Individual	Mean number of correction trial per session	Standard deviation
Premise pairs	Daenerys	8,47	10,13
	Dion	12,27	14,47
	Octo	8,82	8,54
	Starr	7,93	10,00
	MEAN	**9,33**	**10,65**
Mixed pairs	Daenerys	13,33	2,77
	Dion	7,26	2,43
	Octo	8,53	2,34
	Starr	7,03	1,56
	MEAN	**9,04**	**2,94**

Supplementary Table S3. Detail of the learning and test (lines) performances at the individual level (columns) for a more visual comparison. The two individuals who did not reach the learning criteria for all training steps are not included.

	Starr	Dion	Daenerys	Octo	Group mean
total sessions to criteria	39	43	54	69	51.25
% success at the end of the training	78.3	90	72.5	80.8	80.42
% AB	87.5	87.5	83.3	95.8	88.53
% BC	79.2	95.8	62.5	58.3	73.95
% CD	70.8	83.3	62.5	83.3	74.97
% DE	58.3	83.3	66.7	75	70.83
% EF	95.8	100	87.5	91.7	93.75
% success for control trials (36 trials)	100	100	88.89	97.22	96.53
% success for inference trials (12 trials per pair)	80.56	94.45	72.23	77.78	81.26
% BD	91.67	91.67	58.33	75	79.17
% BE	91.67	100	91.67	91.67	93.75
% CE	58.33	91.67	66.67	66.67	70.84

Supplementary Material S2: Controls of confounding factors on choice behavior and performance during test sessions

1) <u>The reinforcement ratio of the items resulting from the training stage did not influence the performance in test trials</u>

We measured the reinforcement ratio of the items involved in the inference trials (B, C, D and E) to observe if there would be any effect on the transitive response (Lazareva et al. 2004, Daniels et al. 2014). The reinforcement ratio of each item X was calculated as the total number of trials presented during the training (including correction sessions) where X was reinforced, divided by the total number of trials presented with X (reinforced and non-reinforced). For example, the reinforcement ratio of B was calculated as follows: $R_B=N_{BC}/(N_{AB}+N_{BC})$. R_B was used to calculate the differential of reinforcement ratios of the two items involved in each inference trial type. For example, the differential reinforcement ratio in BD was calculated as follows: $D_{BD}=R_B-R_D$. The higher D_{BD} is, the more the individual would have been reinforced for item B in comparison to item D. We measured the correlation between D and the TI performance (number of successful trials) for each trial type (BD, BE or CE) for each individual.

(a)	Item B	Item C	Item D	Item E
Starr	0.538	0.514	0.464	0.388
Octo	0.585	0.474	0.469	0.513
Dion	0.556	0.495	0.462	0.364
Daenerys	0.617	0.528	0.448	0.284
Median Ratio	0.571	0.505	0.463	0.376

(b)	Pair	Mean % of success	Differential reinforcement ratio
Starr	BD	91.7	0.074
	BE	100	0.150
	CE	58.3	0.126
Octo	BD	75	0.116
	BE	100	0.072
	CE	66.7	-0.039
Dion	BD	91.7	0.094
	BE	100	0.192
	CE	91.7	0.131
Daenerys	BD	58.3	0.169
	BE	91.7	0.333
	CE	66.7	0.244

Supplementary Table S4. **(a)** Detail of the reinforcement ratio for each term of the series (except the extremity items) at the end of the training stage, at the individual level. **(b)** Detail of the TI performance and the differential reinforcement ratio for each nonadjacent pair (inference trials) at the individual level. The performance is indicated as the percentage of success for the 12 trials for each pair. The differential reinforcement ratio is calculated as, with the example for the pair BD: $D_{BD}=R_B-R_D$ with $R_B=N_{B+C-}/(N_{A+B-}+N_{B+C-})$ and $R_D=N_{D+E-}/(N_{C+D-}+N_{D+E-})$.

At the group level, the differential of the reinforcement ratio of the items cannot explain the performance (Spearman correlation test, S=267.78, p=0.8441, rho=0.0637). At the individual level, this result is confirmed except for Daenerys whose TI performance increases with the differential ratio reinforcement.

In addition, we measured whether the number of presentations of an item during the whole training procedure and its reinforcement history could have an effect on the performance of individuals when this item was presented in an inference trial. First, we measured the number of presentations of each item (B, C, D, E) during the whole training procedure, either (1) in a pair in which this item is the reinforced item (RI; e.g., B in BC), or (2) in a pair in which this item is not the reinforced item (NRI; e.g. B in AB). Second, we detailed the individual performance for each inference trial, either (1) when the trial included the item as the prior item (PI; e.g. BD for the item B), or (2) when the trial included the item as the non-prior item (NPI; e/g/ BD for the item D). We found neither an effect of the total number of presentations of an item during training on the TI performance (F=0.08847, p=0.7676), nor an interaction with the priority of this item in the inference trial (F=0.0003911, p=0.9856; anova permutation model analysis).

2) The configuration of the previous pair trial did not influence the choice behaviour in the following test trial

We tested whether the relationship between the configuration of the previous training pair trial H_{n-1} and the configuration of the test trial T_n would have an influence on the success at this test trial T_n. This relationship was described in terms of whether H_{n-1} and T_n had the same item or not (we analyzed it for the prior and the non-prior item); if this item was presented on the same

side or not; and whether the prior item was presented on the same side in both (H_{n-1} and T_n) or not. None of these parameters did significantly influence the performance in test trials, either control or inference trials (anova permutational model; same item: $F=0.1566$, $p=0.9316$; same side if same item: $F=0.2944$, $p=0.8114$; same side prior item: $F=0.2318$, $p=0.7647$).
